# Cropland type shapes arbuscular mycorrhizal fungi (AMF) population density and species diversity in Northwest Ethiopia

**DOI:** 10.1371/journal.pone.0356876

**Published:** 2026-08-28

**Authors:** Dessie Kassew, Jemal Yimer, Marcela Claudia Pagano, Fassil Assefa, Laith Khalil Tawfeeq Al-Ani, Kemachew Ayele, Belay Berza Beyene

**Affiliations:** 1 Department of Biology, College of Natural and Computational Sciences, Debre Markos University, Debre Markos, Ethiopia; 2 Department of Microbial Sciences and Genetics, School of Biological Sciences, College of Natural and Computational Sciences, Addis Ababa University, Addis Ababa, Ethiopia; 3 Department of Botany, Federal University of Minas Gerais, Belo Horizonte, Brazil; 4 School of Biology Science, University Sains Malaysia, Minden Pulau Pinang, Malaysia; Universidade de Coimbra, PORTUGAL

## Abstract

AMF play critical function in soil fertility and plant nutrition, especially in absorption and translocation of immobile nutrients. Farm and soil fertility management activities enhance crop yield; however, they either positively or negatively impact the AMF population and diversity. This study was aimed at assessing effects of farm and soil fertility management activities on AMF population density, species diversity and richness in North West Ethiopia. Soil samples were obtained from teff (*Eragrostis tef*) and maize (*Zea mays*) croplands. AMF spores were extracted, quantified, and identified. Average AMF spore population was varied significantly (p < 0.05) across districts, among specific sampling locations, and between crop types. Controls exhibited a higher average AMF population density (63.33 spores per 100 g dry soil) as compared to the average AMF population density of croplands (47.59 spores per 100 g dry soil). Maize croplands exhibited the highest average spore densities, up to 112.67 spores per 100 g dry soil and harboring a higher average AMF population density (66 spores per 100 g dry soil) as compared to the teff croplands (30 spores per 100 g dry soil). A total of 14 AMF morphotypes belonging into three genera were identified. *Acaulospora* and *Pacispora* were found dominant, while *Acaulospora myricarpa* and *Pacispora franciscana* were dominant species. Farm and soil fertility management practices decreased AMF population density and species diversity. Maintaining AMF population density and species diversity is needed to enhance soil health and crop productivity in the era of climate change to promote sustainable agriculture.

## 1. Introduction

Arbuscular mycorrhizal fungi (AMF) establish a mutual association with the roots of terrestrial plants. They play a critical role in enhancing health and productivity of agricultural ecosystems. These fungi also establish symbiotic associations with important cereal crops including economically vital crops such as teff (*Eragrostis tef*) and maize (*Zea mays*) [1]. The AMF-plant partnerships significantly enhance plant nutrient acquisition, particularly weakly mobile soil nutrients like phosphorus, nitrogen, potassium, and magnesium in agricultural ecosystems [2–5]. In addition to improving nutrient uptake, AMF enhance soil water retention capacity, improve plant health, and enhance resilience to numerous environmental stressors including drought, acidity and salinity stresses, and the presence of harmful soil compounds [1,2,6–8]. Moreover, the AMF contribution to the formation of stable soil structures, via glomalin synthesis is well established [1,6]. AMF often lead to healthier and more vigorous plant growth, increased biomass and yields even on nutrient-poor soils [9].

The AMF hyphae extend far beyond the plant’s root system, providing extensive surface area for nutrition absorption and translocation. AMF are more efficient in absorption and translocation of phosphorus (P), especially when it exists in less available forms in the soil. This is done by secreting enzymes and acidifying the plant rhizosphere [1,5]. More importantly, AMF form a common mycorrhizal network among nearby plant species which may be the same or different species and thereby share limiting nutrients among the nearby plants [10].

Several reports revealed that soil fertility management practices like tillage, intensive soil fertilization and pest management activities lead to reduced soil biodiversity including microbial diversity [4,11,12]. To mitigate and reduce the negative impacts of soil fertility and pest management practices, an alternative soil fertility management practices like organic farming practices were recommended [12]. The organic farming practices are well known for their high crop yield, favoring sustainable soil biodiversity [13,14]. Organic farming improves soil fertility, soil organic carbon content, and soil microbial diversity [15–17]. However, chemical fertilizer and pesticide application is still a common practice in agricultural ecosystems across the globe.

In the last ten years, there have been intensive chemical fertilizer and pesticide application practices in the croplands in East Gojam Zone, Amhara National Regional State. However, the impacts of these soil fertility and pest management approaches on AMF population density and species diversity in teff and maize croplands has been insufficiently studied in Ethiopia in general and East Gojam zone, north west Ethiopia in particular. Therefore, there is scarcity of detailed scientific information and study concerning the effects of farm and soil fertility management practices on AMF population density and species diversity in the study areas. Here we forward these two hypotheses: (1) frequent soil disturbance disrupts AMF hyphal networks and reduces their population density as compared to non-cultivated controls; (2) maize croplands support a higher AMF population density and species diversity compared to the teff croplands. Therefore, this study was aimed at evaluating effects of the farm and soil fertility management practices on AMF population density, species diversity, community composition and species richness in teff and maize croplands in the East Gojjam Zone, Northern Ethiopia.

## 2. Materials and methods

### 2.1. Area of study

This research was done in three districts: Aneded, Awabel and Gozamin in the East Gojjam Zone Amhara national regional state, North West, Ethiopia. These districts are located approximately 265 kilometers southeast of Bahir Dar, the capital of the Amhara National Regional State and 300 kilometers from the Ethiopia’s capital, Addis Ababa. The geographical coordinates of the sampling districts range between 37^0^42’00” to 37^0^45’30” east longitude and 1^00^17’00” to 1^00^21’30” north latitude. The elevation of the sampling locations range between 2350–2500 m.a.s.l. Sampling districts receive an average annual rainfall of 1380 mm, with the average lowest and highest temperature of 12 °C and 25 ^0^C, respectively. The agro-ecology of the sampling districts range from kola to wurch. The major crops cultivated in the sampling districts include: teff, wheat, barley, maize, engido, beans, peas, lupins, etc.

### 2.2. Preliminary survey and soil sampling

A preliminary survey was conducted on the farm and soil fertility management practices in the sampling districts. During preliminary survey, information about previous cropping history, chemical fertilizer application, pesticide application and lime amendment history (Supplementary information 1) were carried out. The soil samples were obtained from the three districts, namely Gozamen, Aneded and Awable districts of the East Gojam Zone. These districts were selected based on their well known teff and maize production. At each district, farmlands were randomly selected. A total of 14 croplands (7 teff and 7 maize) soil samples were collected. The size of the croplands range between 0.5 hectare and 2.5 hectares. During soil sampling, a farmland was divided into five sampling points: North, South, East, West, and the Center [1]. The five sampling points were cleaned and dug up 30 cm for soil sampling. The soil samples were pooled into one composite sample. Control samples were obtained from the nearby farmlands, which were left without agricultural practices during the sampling seasons. Soils were collected in sterile polythene bags. The sample collection was carried out during dry season from October to December, 2023. The samples were carried to the Debre Markos University, Department of Biology, Mycology and Microbiology laboratory and Addis Ababa University, Department of Microbial Cellular and Molecular Biology, Applied Microbiology Laboratory for further analysis. The 1 kg soil was divided into 2 subsamples (500 g each), for AMF spore extraction and for soil parameter analysis. This study was reviewed and approved by Ethical review committee of Department of Biology, Debre Markos University. An informed consent was obtained from all the farmland owners for participating in this study.

### 2.3. The soil parameters

Soil samples were processed and analyzed at two primary locations: the Engineering Corporation of Oromia (ECO) Soil, Water, and Plant Laboratory in Addis Ababa, and the Mycology and Microbiology Laboratory at Debre Markos University, Ethiopia. Prior to soil chemical analysis, soil sub-samples were homogenized through grinding and passed through a 2 mm mesh sieve. The following parameters were then determined: Soil pH was measured in a 1:2.5 (w/v) soil-to-water suspension, following the protocol by Carter and Gregorich [18]. Soil organic carbon (SOC) was quantified using the chromic acid titration method described by Walkley and Black [19]. Total nitrogen (TN) was determined via the Kjeldahl digestion method, as outlined by Hinds and Lowe [20](1980) and Available phosphorus (P) was extracted and measured according to the Olsen method [21].

### 2.4. Arbuscular mycorrhizal fungi spore extraction

Arbuscular mycorrhizal fungal (AMF) spores were isolated from the soil using a modified wet sieving and decanting technique, as detailed by Gerdemann and Nicolson [22]. A 100 g of air-dried soil samples were processed in triplicate. Each sample was submerged in 500 mL water for approximately 1–2 hours. After manually breaking down larger soil aggregates and allowing the heavy particles to settle, the resulting liquid was poured through a stacked series of sieves with mesh sizes of 500 µm, 212 µm, 106 µm, and 45 µm. The sieving process continued with tap water until the runoff was transparent. Materials caught in the 500 µm sieve were examined for the presence of spore clusters or sporo-carps; spores found were moved to the 45 µm sieve. Similarly, residues from 212 µm and 106 µm sieves were rinsed and consolidated into the 45 µm sieve. The collected materials were then moved to 15 mL Falcon tubes and subjected to centrifugation at 2000 rpm for 5 minutes. After discarding the supernatant, the pellets were re-suspended in a 50% (w/v) sucrose solution and centrifuged again for 3 minutes at 2000 rpm. The resulting supernatant was captured on a 45 µm sieve, washed thoroughly to eliminate residual sugar, and transferred to a 90 mm gridded Petri dish. Spore densities were determined using a stereomicroscope (NOVEX, ISO 1006) at 40X magnification, following the identification and counting guidelines provided by INVAM. This entire procedure was performed three times, utilizing a total of 300 g of soil.

### 2.5. AMF identification and characterization

Individual AMF spores were isolated under a dissecting microscope using a micropipette. For permanent preservation, spores were mounted on glass slides using either Polyvinyl-Lactic acid-glycerol (PVLG) or a 1:1 (w/v) mixture of PVLG and Melzer’s reagent, following the protocols established by the Glomeromycota database (https://maarjam.ut.ee/). Initial observations and digital imaging were performed using a compound microscope (Olympus-BX51) at magnifications of 20x, 40x, and 100x. Subsequently, the spores were crushed to reveal internal wall layers and allowed to air-dry at room temperature for two weeks. These crushed specimens were then re-examined and photographed under the same magnification range (20x–100x) to document detailed morphological features. Taxonomic identification to the genus and species levels was conducted based on diagnostic physical characteristics, including: spore coloration and surface ornamentation, wall structure and thickness and the morphology of the subtending hyphae. These features were cross-referenced with official descriptions from INVAM and the Glomeromycota online repository, utilizing the classification keys proposed by Schenck [23]. This identification work was carried out through collaboration between the Applied Microbiology Laboratory at Addis Ababa University (Department of Microbial, Cellular, and Molecular Biology) and the Department of Biology at the Federal University of Minas Gerais, Brazil.

### 2.6. AMF population density and species richness

To evaluate the AMF community structure across the various sampling districts and farmland locations, the following ecological parameters were employed: 1) abundance and richness: population density (SD) was calculated as the total number of AMF spores identified 100 g^-1^ dry soil. Species richness (S) was defined as the total count of distinct AMF species identified within a specific sampling location or district. 2) diversity Indices: the Shannon–Wiener Index (H’) was used to assess species diversity, calculated as: H’ = Σ (ni/n)ln(ni/n), where: ni = number of individuals of species n, and n = number of all individuals of all species. The Simpson’s dominance index (D) was calculated using the formula: $D=\Sigma \frac{ni(ni-\text{1})}{N(N-\text{1})}\;$ where ni = the total number of individuals of a single, specific species (i), N = the total number of all individuals of all species in the sample, Σ = the sum of the calculations for each individual species. 3) Occurrence and dominance: the Frequency of occurrence (FO) represents the percentage of samples in which a specific genus or species was detected, was calculated according to Fernandes et al. [24]. That is ([the number of samples in which a given species or genus was isolated/ the total number of samples] ×100). The Isolation Frequency (IF) served as a measure of species dominance. The following the classification by Chen et al. [25] was used for a species or genera to categorize based on their IF values: Accordingly, dominant: IF, IF ≥50%, common: 10% <IF <50% and Rare: if, < 10%.

### 2.7. Statistical analysis

Average AMF spore densities were analyzed using a one-way ANOVA in SAS (version 9.4) to evaluate significant differences across sampling districts, locations, and crop types. Prior to conducting tests, the underlying assumptions of analysis of variance (ANOVA) such as normality of the individuals, homogeneity of variances across groups were verified. Where significant effects were found, LSD Test was employed for mean separation at a significance level of p < 0.05.

## 3. Results

### 3.1. The preliminary survey of farm and soil fertility management practices

The soil types of the croplands included in this study were black soils (Vertisols) with no lime amendment history in major teff growing districts (Aneded and Awabel). Red soils (Nitisols) with no lime amendment history were recorded in major maize growing district (Gozamin). The previous cropping history of these croplands also exhibited crop rotation practices (S1 Table). About 43% of the teff croplands exhibited crop rotation practices with legumes (vetch) in the previous year (S1 Table). Similarly, about 57% of the maize croplands showed crop rotation with different crop such as potatoes and wheat (S1 Table).

The survey results further revealed that farm and soil fertility management practices have been involving increased and repetitive chemical fertilizer and pesticide applications in all the sampling locations and districts in the past ten years.

### 3.2. Soil properties

The physicochemical characteristics of soils were varied across sampling districts. Soil pH at the sampling districts was ranged from moderately acidic (5.7 at Awabel district) to slightly acidic (6.52 at Gozamin district) (Table 1).

**Table 1:** Physicochemical properties of soils in the sampling districts.

Sampling Districts	pH	Organic carbon (%)	Organic matter (%)	Total nitrogen (%)	Av.P (ppm)
Awabel	5.7	2.00	3.44	0.17	**51.36**
Aneded	5.93	2.42	4.16	0.17	**57.34**
Gozamin	6.52	1.4	2.41	0.17	**64.78**

The soil organic carbon (OC) content was the highest at Aneded (2.42%) followed by (2.00%) at Awabel district, while the Gozamin district exhibited the lowest (1.4%) soil organic carbon. Correspondingly, the highest soil organic matter (OM) (4.16%) was recorded at the Aneded district, followed by (3.44%) at the Awabel district and the Gozamin district exhibited the least (2.41%). The total nitrogen (TN) was similar (0.17%) across sampling districts. In a similar context, the highest available phosphorus (P) (64.78 ppm) was recorded at the Gozamin district, while the least available P (51.36 ppm) was recorded at the Awabel district (Table 1).

### 3.3. AMF spore densities across the sampling locations

We have recorded significantly (p < 0.05) different average AMF spore population across sampling locations at Aneded district under teff cropland. The sampling location 4 (S4) exhibited the highest average AMF population density (16.67 spores per 100 g dry soil), whereas sampling location 2 (S2) exhibited the lowest average AMF population density (11.33 spores per 100 g dry soil) (Table 2). The control samples exhibited much higher average AMF population density (50.33 spores per 100 g dry soil) compared to the Teff cropland soils (Table 2).

**Table 2:** Sampling locations, GPS coordinates and average AMF population density of Teff croplands at Aneded District.

Sampling location	GPS coordinates		Altitude (m.a.s.l)	Population density per 100 g dry soil
	Longitude	Latitude		
S1	37^0^ 43’32.58”	10^0^ 19’00.45”	2476	12 ± 2.95d
S2	37^0^43’32.90”	10^0^19’00.34”	2477	11.33±1.7e
S3	37^0^43’32.97”	10^0^19’01.30”	2478	14.67± 4.78c
S4	37^0^43’33.01”	10^0^19’01.90”	2480	16.67± 3.3b
Control	37^0^43’33.90”	10^0^19’56.22”	2482	50.33± 13.1a

Values are mean±SD and are expressed as means of triplicate experiments. Means with the same letter in the same column are not significantly different at p < 0.05 by LSD test.

Similar to the Aneded district, the average AMF spore population was significantly (p < 0.05) varied across sampling locations at Awabel district under teff cropland. The highest average AMF spore population was recorded from the sampling location 1 (S1) (68.67 spores per 100 g dry soil), whereas the lowest average AMF population density (8.33 spores per 100 g dry soil) was recorded from sampling location 3 (S3) (Table 3). In the same context, the control sample exhibited numerically higher average AMF population density (69.67 spores per 100 g dry soil) compared to the Teff cropland soils (Table 3).

**Table 3:** Sampling locations, GPS coordinates and average AMF population density of teff croplands at Awabel district.

Sampling locations	GPS coordinates		Altitude (m.a.s.l)	Population density per 100 g dry soil
	Longitude	Latitude		
S1	37^0^50’33.07”	10^0^15’57.79”	2372	68.67 ± 16.05a
S2	37^0^50’33.13”	10^0^15’57.74”	2370	41.00 ± 3.56b
S3	37^0^50’33.09”	10^0^15’57.32”	2371	38.33 ± 2.87c
Control	37^0^50’33.25”	10^0^15’57.84”	2374	69.67 ± 13.05a

Values are mean±SD and are expressed as means of triplicate experiments. Means with the same letter in the same column are not significantly different at p < 0.05 by LSD test

The average AMF spore population was significantly (p < 0.05) varied across sampling locations at the Gozamin district under maize cropland. The highest average AMF population density (112.67 spores per 100 g dry soil) was recovered from the sampling location 7 (S7), followed by 83 spores per 100 g dry soil from sampling location 2 (S2). However, the lowest average AMF population density (40.67 spores per 100 g dry soil) was recorded from sampling location 1 (S1) (Table 4). The control samples at the Gozamin district exhibited the lowest average AMF population density, 71 spores per 100 g dry soil compared to the maize cropland soils (Table 4).

**Table 4:** Sampling locations, GPS coordinates and AMF population density of maize cropland at Gozamin district.

Sampling locations	GPS coordinates		Altitude (m.a.s.l)	Population density per 100 g dry soil
	Longitude	Latitude		
S1	37^0^43’32.03”	10^0^18’54.03”	2473	40.67 ± 9.18g
S2	37^0^43’32.27”	10^0^18’56.16”	2471	83.00 ± 21.97b
S3	37^0^43’32.45”	10^0^18’56.18”	2472	47.67 ± 7.58f
S4	37^0^43’32.37”	10^0^18’56.15”	2474	62.33 ± 14.82d
S5	37^0^43’32.70”	10^0^18’56.17”	2469	56.00 ± 5.89e
S6	37^0^43’32.77”	10^0^18’55.99”	2475	61.33 ± 2.87d
S7	37^0^43’33.07”	10^0^18’56.32”	2476	112.67 ± 9.74a
Control	37^0^43’33.05”	10^0^18’56.85”	2478	71.00 ± 26.51c

Values are mean±SD and are expressed as means of triplicate experiments. Means with the same letter in the same column are not significantly different at p < 0.05 by LSD test.

**Fig 1:**
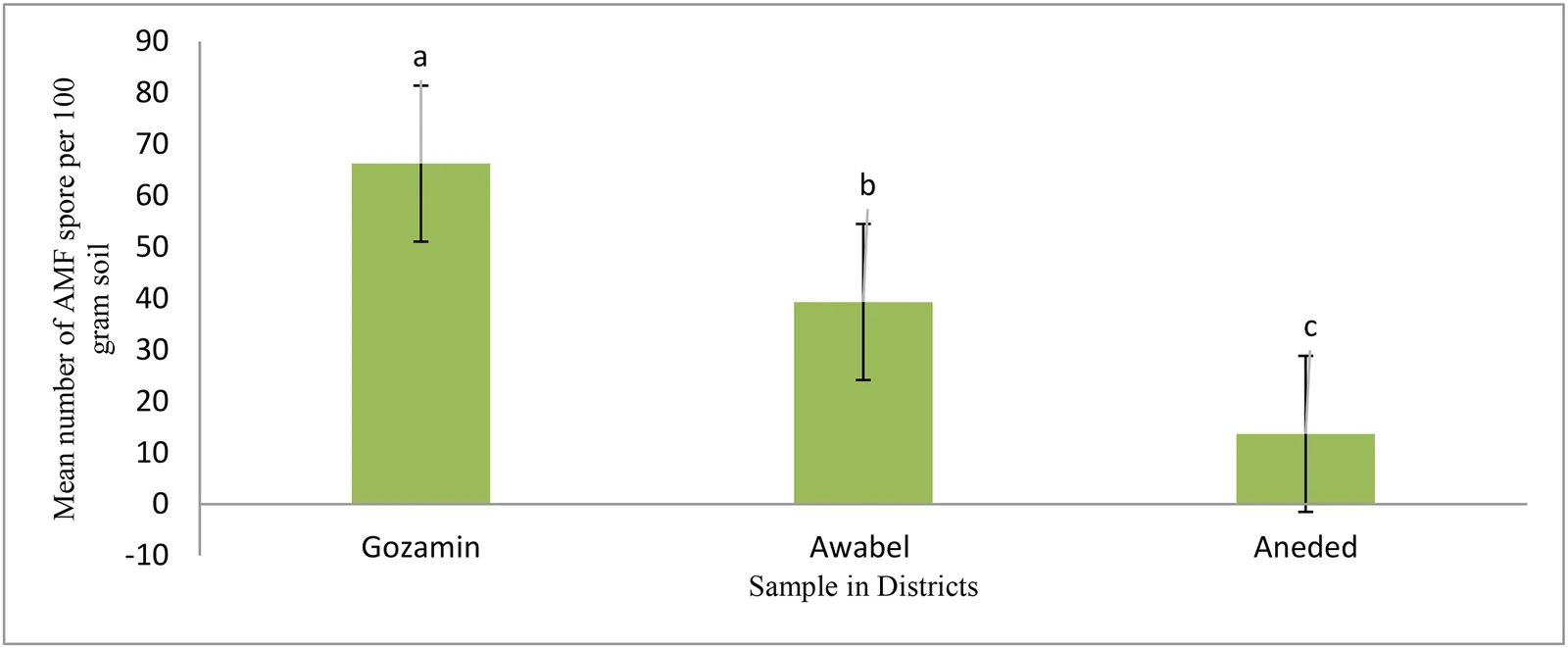
Average AMF spore densities (spores per 100 g dry soils) at district level. Values are mean±SD and are expressed as means of triplicate experiments. Means with the same letter in the same column are not significantly different at p < 0.05 by LSD test.

When comparing districts, the highest average AMF population density (66.24 spores per 100 g dry soil) was recorded from Gozamin under maize croplands followed by 39.33 spores per 100 g dry soil from the Awabel district (Fig 1) from Teff cropland.

**Fig 2:**
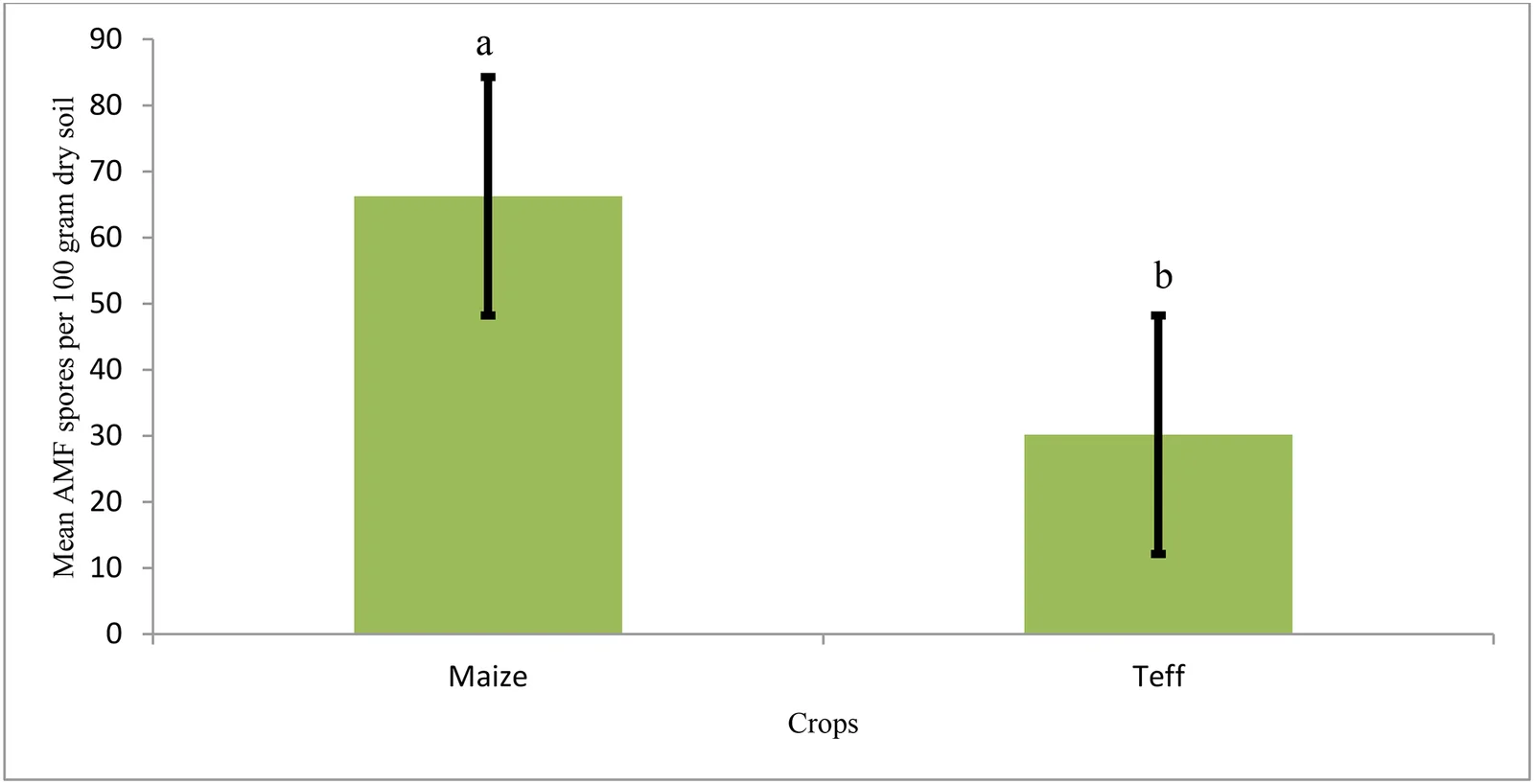
Average AMF spore densities per 100 g dry soil at crops level. Values are mean±SD and are expressed as means of triplicate experiments. Means with the same letter in the same column are not significantly different at p < 0.05 by LSD test.

Similarly, when comparing the average AMF spore densities per 100 g dry soil between crops, the maize crops rhizosphere has exhibited the highest average AMF population density (66 spores per 100 g dry soil), while Teff cropland exhibited about 30 spores per 100 gram dry soil (Fig 2).

**Fig 3:**
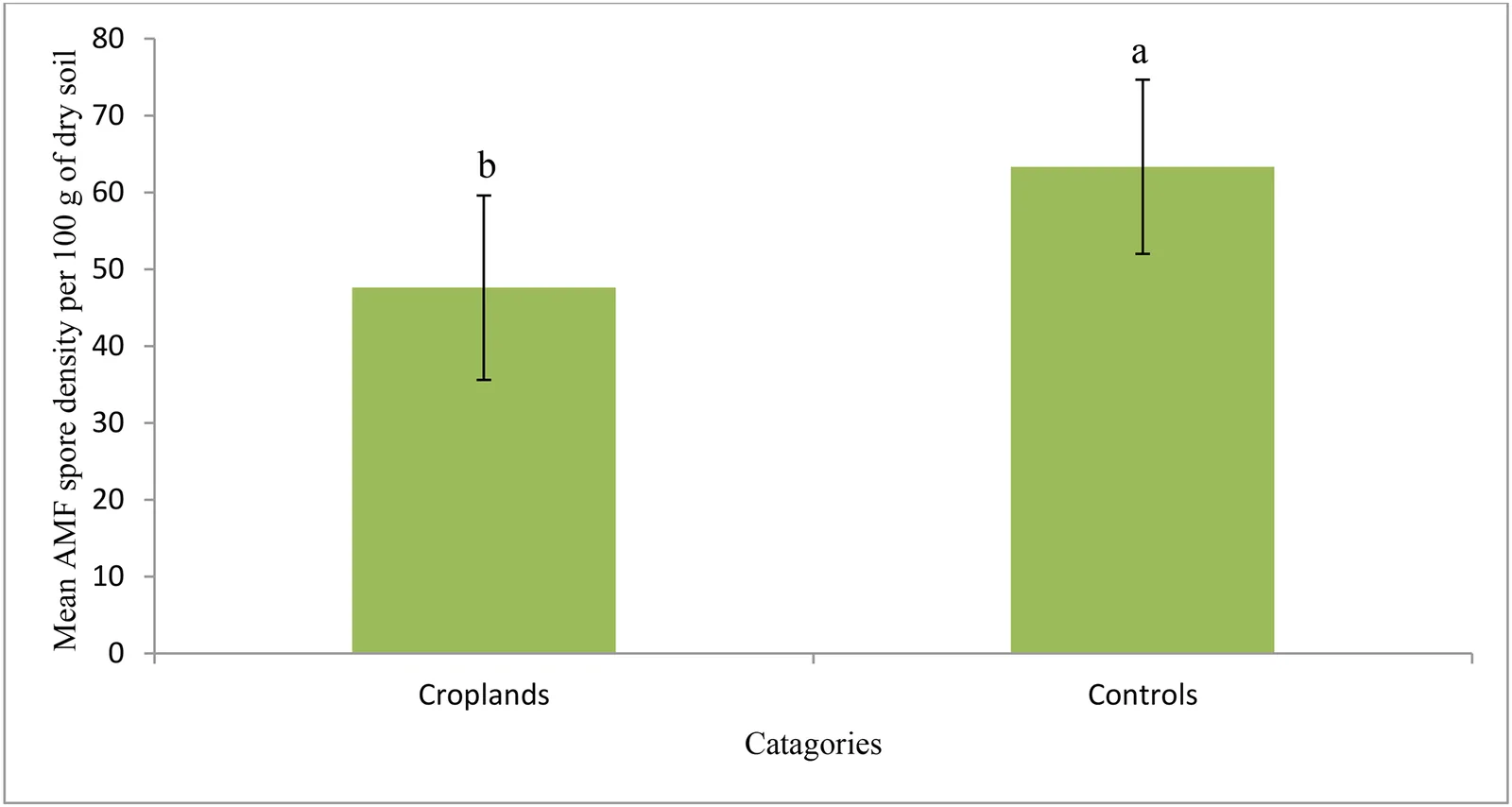
Average AMF spore densities per 100 g dry soil as compared between croplands and controls. Values are mean±SD and are expressed as means of triplicate experiments. Means with the same letter in the same column are not significantly different at p < 0.05 by LSD test.

Similarly, average AMF spore population was significantly (p < 0.05) varied between croplands and controls. Controls exhibited the highest average AMF population density (63.33 spores 100 g dry soil) as compared to the croplands, which exhibited 47.59 spores 100 g^-^ dry soil (Fig 3).

### 3.4. AMF genus and species diversity and abundance

A total of 14 AMF species/morphotypes classified into three genera were recovered. Among the genera, *Acaulospora* and *Pacispora* were recorded dominant, both are recovered from 9 soil samples out of 14 samples (IF; 64.28%) (S2 Table).

**Fig 4:**
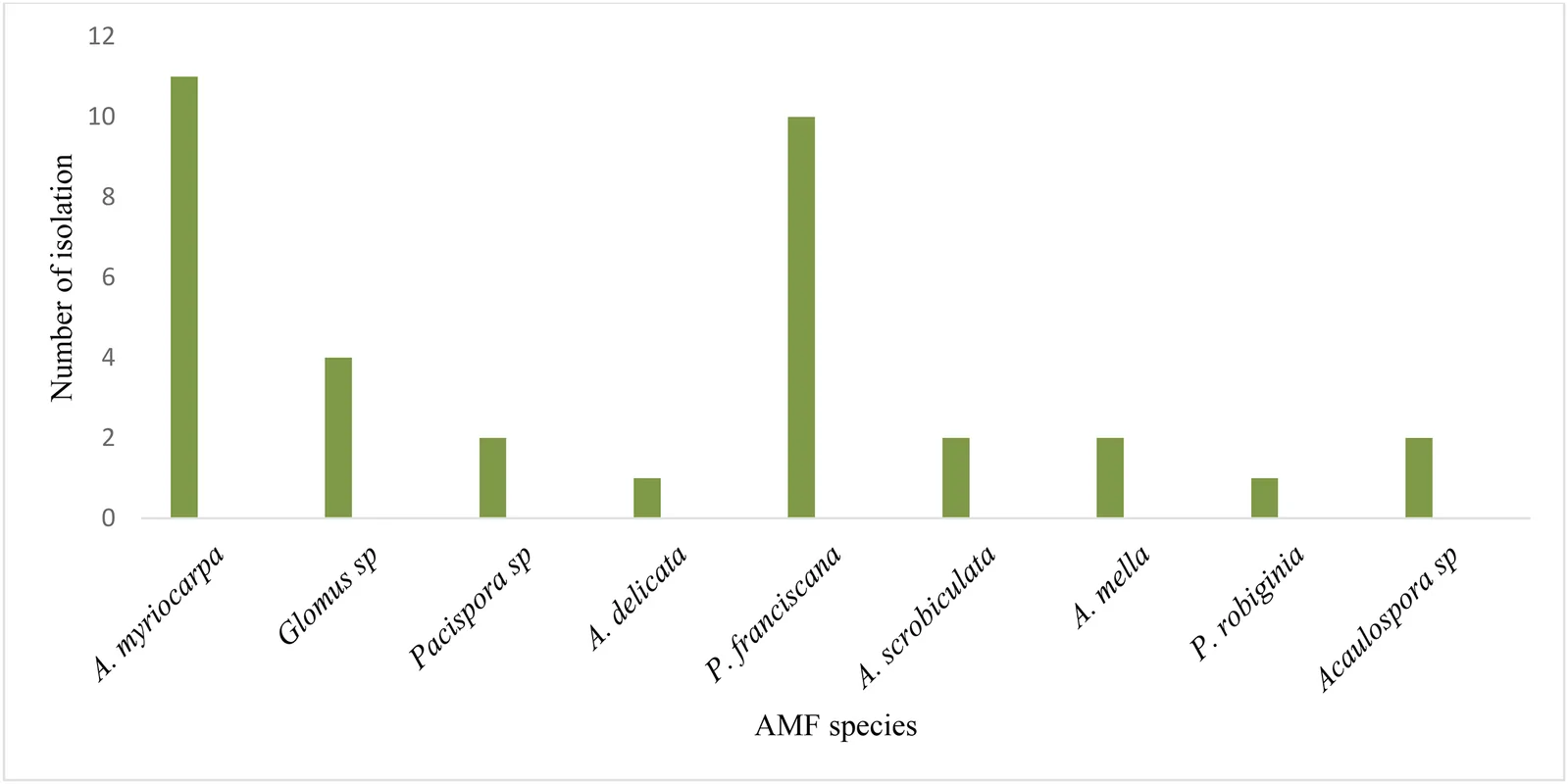
The number of isolations of AMF species.

Genus *Glomus* was categorized as common, which was recovered from 4 soil samples out of 14 samples (IF; 14.28%). At the species level, *Acaulospora myriocarpa* was a dominant species with IF value of 78.57% and RA value of 78.57% followed by *Pacispora franciscana*, having IF value and RA value of 71.43% each (S2 Table). The number of isolations of each AMF species/ morphotypes is presented in Fig 4.

The common species recovered from the sampling distrAMFicts included *Glomus* sp1, *Pacispora* sp1, *Acaulospora scrobiculata*, *Acaulospora mellea*, *Acaulospora* sp1, *Glomus* sp2.

**Table 5:** The isolation frequency (IF), relative abundance (RA) of AMF species.

Number	AMF species	IF (%)	RA (%)	Status
1	*Acaulospora myriocarpa*	78.57	78.57	Dominant
2	*Glomus* sp1	28.57	28.57	Common
3	*Pacispora*sp1	14.28	14.28	common
4	*Acaulospora delicata*	7.14	7.14	Rare
5	*Pacispora franciscana*	71.43	71.43	Dominant
6	*Acaulospora scrobiculata*	14.28	14.28	Common
7	*Acaulospora mellea*	14.28	14.28	Common
8	*Pacispora robiginia*	7.14	7.14	Rare
9	*Acaulospora* sp1	14.28	14.28	Common
10	*Glomus* sp2	28.57	28.57	Common
11	*Glomus* sp3	28.57	28.57	Common
12	*Glomus* sp4	28.57	28.57	Common
13	*Acaulospora* sp2	14.28	14.28	Common
14	*Pacispora* sp2	14.28	14.28	Common

**Fig 5:**
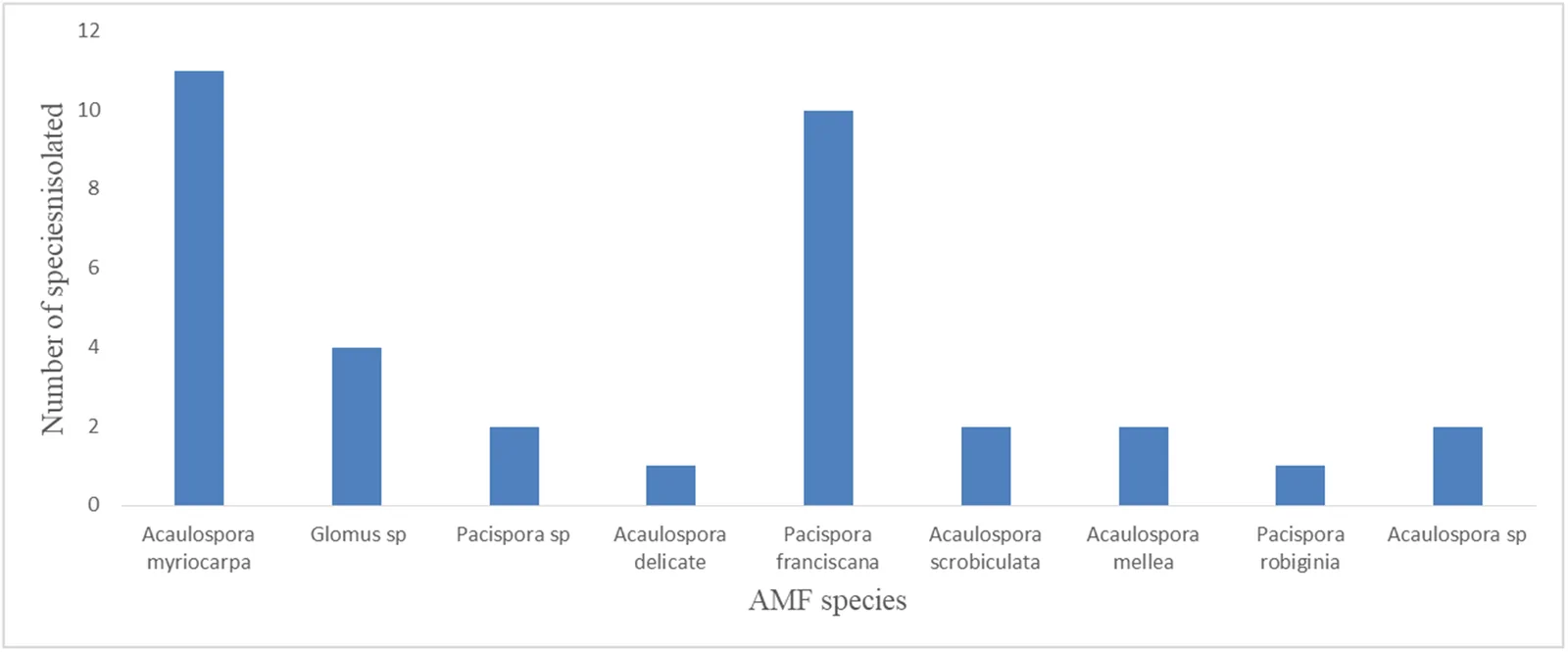
The number of isolations of each AMF species.

*Glomus* sp3, *Glomus* sp4, *Acaulospora* sp2, and *Pacispora* sp2 (Table 5). The rare AMF species identified from the sampling districts were *Acaulospora delicata* and *Pacispora robiginia*. Fig 5 presents AMF species level isolation frequency at the sampling districts.

### 3.5. AMF genus and species richness associated to the crop type

The highest species richness was recorded at Gozamin district. At Gozamin district, from maize cropland, we have recorded three AMF genera and eight species (Table 6). These included; *Acaulospora delicata, Acaulospora mellea, Acaulospora myriocarpa, Acaulospora scrobiculata, Glomus* sp*, Pacispora sp, Pacispora franciscana, Pacispora robiginia*. Similarly, at Awabel and Aneded districts, from teff cropland, three AMF genera comprised of six species were isolated. These species included *Acaulospora* sp*, Acaulospora mellea, Acaulospora myriocarpa, Glomus* sp*, Pacispora* sp*, Pacispora franciscana* (Table 6).

**Table 6:** AMF genus richness, number of each genus and species richness (SR) per crop type at the study districts.

Crop type	Genus name	Species Richness (for the crop)	Breakdown of Species Richness
Maize	*Acaulospora*	8	*Acaulospora delicata* *Acaulospora mellea* *Acaulospora myriocarpa* *Acaulospora scrobiculata* *Glomus* sp *Pacispora* sp *Pacispora franciscana* *Pacispora robiginia*
	*Glomus*		
	*Pacispora*		
Teff	*Acaulospora*	6	*Acaulospora sp* *Acaulospora mellea* *Acaulospora myriocarpa* *Glomus* sp *Pacispora* sp *Pacispora franciscana*
	*Glomus*		
	*Pacispora*		

### 3.6. AMF Diversity indices

AMF genus and species diversity indices varied across the sampling districts and crop types. The Gozamin district, from which maize cropland soil was sampled, exhibited eight AMF species richness (S=8) (Table 7) followed by the Aneded districts showing five species richness (S=5) and with teff crop cultivation. The AMF species diversity indices were also varied among sampling districts and between crop types. The Simpson’s Index of Diversity (1-D) of 0.776 and Shannon diversity Index (H’) of 1.654 were recorded at Gozamin district.

**Table 7:** AMF Diversity Indices at sampling districts.

Location	Crop	Species Richness (S)	Simpson’s Index of Diversity	Shannon Index of diversity
Gozamin	Maize	8	0.776	1.654
Awabel	Teff	3	0.833	1.040
Aneded	Teff	5	0.844	1.504

The highest Index of Diversity (1-D) of 0.844 was recorded at Aneded district under teff cropland followed by the Awabel districts with the Simpson’s index of diversity (0.833) similarly under teff cropland. Similarly, a Shannon diversity Index value of 1.040 and 1.504 were recorded for Awabel and Aneded districts, respectively both under teff cropland (Table 7).

## 4. Discussion

The symbiotic relationship between plants and AM fungi is the most widespread plant-fungus interactions in natural and cropping systems [26]. AMF play vital significance in the establishment of terrestrial plants by highly affecting their growth and development, nutrient absorption and resilience to biotic and abiotic stressors. [4,27]. They improve plant growth, disease resistance, and sustainable crop yield by improving plant performance via enhanced nutrient acquisition by using the extensive extra radical hyphal or mycelia networks [5,10]. However, agricultural, farm and soil fertility management activities like extensive tillage, fertilizer and pesticide usage significantly impaired and reduced AMF population density and species diversity leading changes in AMF community composition [28,29].

We have obtained a significant (p < 0.05) variation in average AMF population density in 100 g dry soil between extensively cultivated teff croplands and uncultivated controls across sampling locations and districts. The average AMF population density from overall teff farms was about 28 spores per 100 g dry soil, while the uncultivated controls exhibited average population density of 60 spores per 100 g dry soil, which is twice higher than the average AMF population density of the teff croplands (Fig 3). Our results are in line with the results of Berza et al., [1], who have recorded average of 26 AMF spores per 100 g dry soil from teff rhizosphere in Enrata and Gozamin districts. Therefore, repetitive cultivations and increased chemical fertilizer and pesticide applications could be possible reasons for reduced AMF population density under teff farmlands compared to the uncultivated controls. This is because repeated cultivations breaks AMF hyphal networks thereby disrupts AMF population proliferation.

Significant variations were observed in AMF spore densities both within the sampling locations and across districts. This is most likely associated to the conventional agricultural practices and farm and soil fertility management approaches [4]. The differences in average AMF population density between uncultivated controls and teff croplands strongly suggests that the farm and soil fertility management practices employed in the teff cultivation were detrimental to AMF spore production. Such practices in our sampling locations and districts include intensive tillage, synthetic fertilizer application (especially P fertilizers) and pesticide use. Such practices are known to disrupt AMF hyphal networks, reduce host root exudation, and alter soil microbial communities, leading to declines in AMF populations [4,10,30–32]. The control samples, presumably representing less disturbed or uncultivated areas, provided a baseline illustrating the potential AMF proliferation capacity under minimal anthropogenic interference. This again shows that in addition to the AMF hyphal network disruption due to continuous tillage, application of chemical fertilizers (P) and pesticide application could reduce AMF spore production and hyphal network formation. This is because when there is sufficient amount of P in the soil due to the chemical fertilizer usage; plants tend to use the available P in the soil than forming symbiosis with the AMF. Moreover, when there is sufficient available in the soil as a result of chemical fertilizer application, plants reduce release of carbohydrates for AMF use leading to reduced AMF population proliferation.

We have also measured a significant difference in average AMF population density between two crops (maize and teff). The maize cropland consistently harbored a higher average AMF population density (66 spores per 100 g day soil) as compared to teff cropland which exhibited average of 30 AMF spores per 100 g dry soil and this is in agreement with our hypothesis. These differences could be attributed to the fact that maize is widely recognized as highly mycotrophic crop, forming extensive symbiotic relationships with AMF [29]. Maize’s coarse root system provides ample physical space for colonization and delivers high carbon allocation necessary for extensive fungal growth and sporulation [33]. The observed average AMF population density (66 spores per 100 g dry soil) is consistent with levels reported in moderately managed maize fields in the tropics, where high root biomass ensures abundant inoculums [34]. In the contrary, teff is often grown in fields which are subject to very intense annual tillage, including manual crushing of soil aggregates and frequent mono-cropping. Continuous cultivation and associated extensive soil disturbances significantly suppress the AM fungal spore bank, leading to the lower densities [35,36]. These shows how crop type choice, and farm management practices significantly influence AMF population density in the agricultural ecosystems.

In the present study, we have identified a total of 14 AMF species/morphotypes belonging into three genera. *Acaulospora* and *Pacispora* were dominant genera and *Acaulospora myriocarpa* and *Pacispora franciscana* emerged as dominant species. This observation indicates that these genera and species are well adapted to the prevailing conditions and agricultural systems in the study area. The presence of specific dominant genera and species reflect long-term adaptation to specific soil types, climate conditions, and agricultural practices at the sampling locations and districts. Most recently Berza et al [1] have recovered 35 AMF species/morphotypes from wheat, Barley and teff rhizosphere at Gozamin, Enrata and Debre Elias districts of East Gojam Zone, North West Ethiopia. In their study Berza et al. [1] have reported that *Pacispora* was dominant genus and *Acaulospora* was common genus.

In general, the study of diversity of species and AMF community by spore analysis has been regarded as a standard protocol in the detection of effects of agricultural managements on AMF communities [37]. However, the diversity study based solely on spore analysis does not always work as reliable data, as they are often subjected to overestimate and underestimate the true AMF diversity due to the variation of environmental factors, which are able to affect spore formation [38,39]. It is suggested that diversity of AMF must comprise study of soil and root samples and apply of DNA based techniques to overcome any bias and have true population composition at any situation. Therefore, there is now consensus that AMF spore community based analyses does not seem to provide an adequate representation of the active symbiosis, i.e., Functional fungal parts located in and outside root [39,40].

The dominance of *Acaulospora* and *Pacispora* is noteworthy. *Glomus* is often considered a ubiquitous genus, commonly or dominantly found across diverse ecosystems and agricultural settings [41]. Sasvari and Posta [26], have identified that all of sequence types belonging to the *Glomus* clade in a long term maize mono-cropping system. The relatively lower isolation frequency of *Glomus* to the common status in this study could be attributed to the specific environmental conditions or farm and soil fertility management practices which might favor *Acaulospora* and *Pacispora* species. According to Sharmah et al. [42], species from *Acaulospora* are often found in disturbed soils and can tolerate a range of environmental stressors, including low pH and nutrient-poor conditions. The dominance of *Acaulospora* is widely associated with environments characterized by low soil pH (acidity), low soil fertility, or high soil moisture content [30,43]. The soils in our sampling locations comprised of nutrient-leached Nitisols and/or highly weathered soils prone to acidity. The local soil chemistry is also determinant of AMF community structure, overriding the host plant’s ability to select for other genera like *Pacispora* and *Acaulospora*. The higher stress tolerance of *Acaulospora* and *Pacispora* allowed them to thrive where the more sensitive *Glomus* species struggle, regardless of whether the field is planted with maize or teff.

Species richness analysis revealed that maize cultivation in Gozamin district supported the highest number of AMF species (8 species), encompassing all three identified genera. However, teff cultivation supported 6 species, although all three genera were recovered. This further supports the idea that crop types and associated management practices, influence AMF diversity. Maize’s extensive root system and longer growth cycle compared to teff might have provided more stable and diverse niche for AMF [44]. The higher AMF species diversity in maize cropland is supported by the idea that high-biomass and vigorous host plants offer a greater variety of ecological niches and nutrient sources, thus supporting a richer AMF community [34]. However, relatively lower diversity in Teff cropland aligns with studies demonstrating that sustained high-intense monoculture and tillage depletes the soil’s AMF inoculum pool, leaving behind only the most resilient species [36].

The diversity indices further strengthened the differences in the AMF population density and species diversity. The maize cropland exhibited the highest Shannon Index (H’ = 1.654) and species richness (S=8), indicating a diverse and relatively evenly distributed AMF community was supported compared to the teff croplands.

## 5. Conclusion

A higher AMF spore density and species diversity was recorded in maize croplands compared to the teff croplands. The higher average AMF spore density in the control samples highlights the impacts of farm and soil fertility management practices on AMF population density and species diversity and the need for adopting AMF friendly agricultural practices. Promoting farm and soil fertility management approaches that reduce soil disturbance, optimize nutrient management, and diversify cropping systems could be crucial for harnessing the ecological benefits of AMF for sustainable maize and teff based agriculture in the study region.

## Supporting information

S1 Table The preliminary information on the soil types, previous cropping history and chemical fertilizer and pesticide application in the sampling locations and districts. (DOCX)

S2 Table The genus-level Abundance, Isolation Frequency, and Dominance of AMF at study districts. Appendix table 3 the species-level Abundance, Isolation Frequency, and Dominance of AMF at the sampling districts.
(DOCX)

S3 Table The species-level Abundance, Isolation Frequency, and Dominance of AMF at the sampling districts. (DOCX)

S1 File Farm and soil fertility management practices survey information collection tools from Teff and maize croplands at Aneded, Awabel and Gozamin districts. (DOCX)
