# Supplementary material for: Cropland type shapes arbuscular mycorrhizal fungi (AMF) population density and species diversity in Northwest Ethiopia
Source: PLoS One. 2026 Aug 28;21(8):e0356876. doi: 10.1371/journal.pone.0356876 (PMC13524280; doi:10.1371/journal.pone.0356876)
Supplement: Supplementary file 1 [file pone.0356876.s001.docx]

S1

| Sample number | Districts | Region | Soil type | Crop type | previous crop | Liming history | N (46-92 kg/ha) and P (20-30kg/ha) fertilization | cropping season | Pesticide application | Sowing practice |
| --- | --- | --- | --- | --- | --- | --- | --- | --- | --- | --- |
| 1 | Aneded | Amhara | Black | Teff | Vetch | None | Yes, in the last ten years | Rain feed | Yes | Traditional sowing |
| 2 | Aneded | Amhara | Black | Teff | Teff | None | Yes, in the last ten years | Rain feed | Yes | Traditional sowing |
| 3 | Aneded | Amhara | Black | Teff | Teff | None | Yes, in the last ten years | Rain feed | Yes | Traditional sowing |
| 4 | Aneded | Amhara | Black | Teff | Vetch | None | Yes, in the last ten years | Rain feed | Yes | Traditional sowing |
| 5 | Awabel | Amhara | Black | Teff | Teff | None | Yes, in the last ten years | Rain feed | Yes | Traditional sowing |
| 6 | Awabel | Amhara | Black | Teff | Vetch | None | Yes, in the last ten years | Rain feed | Yes | Traditional sowing |
| 7 | Awabel | Amhara | Black | Teff | Teff | None | Yes, in the last ten years | Rain feed | Yes | Traditional sowing |
| 8 | Gozamin | Amhara | Red | Maize | Potato | None | Yes, in the last ten years | Rain feed | Yes | Line sowing |
| 9 | Gozamin | Amhara | Red | Maize | Maize | None | Yes, in the last ten years | Rain feed | Yes | Line sowing |
| 10 | Gozamin | Amhara | Red | Maize | Wheat | None | Yes, in the last ten years | Rain feed | Yes | Line sowing |
| 11 | Gozamin | Amhara | Red | Maize | Wheat | None | Yes, in the last ten years | Rain feed | Yes | Line sowing |
| 12 | Gozamin | Amhara | Red | Maize | Maize | None | Yes, in the last ten years | Rain feed | Yes | Line sowing |
| 13 | Gozamin | Amhara | Red | Maize | Potato | None | Yes, in the last ten years | Rain feed | Yes | Line sowing |
| 14 | Gozamin | Amhara | Red | Maize | Wheat | None | Yes, in the last ten years | Rain feed | Yes | Line sowing |
