# Supplementary material for: Cropland type shapes arbuscular mycorrhizal fungi (AMF) population density and species diversity in Northwest Ethiopia
Source: PLoS One. 2026 Aug 28;21(8):e0356876. doi: 10.1371/journal.pone.0356876 (PMC13524280; doi:10.1371/journal.pone.0356876)
Supplement: Supplementary file 2 [file pone.0356876.s002.docx]

S2

| Genus | Number of samples from which the genus isolated | Isolation Frequency (IF) % | Genera status |
| --- | --- | --- | --- |
| *Acaulospora* | 9 | 64.28% | Dominant |
| *Pacispora* | 9 | 64.28% | Dominant |
| *Glomus* | 4 | 28.57% | Common |
