# Supplementary material for: Cropland type shapes arbuscular mycorrhizal fungi (AMF) population density and species diversity in Northwest Ethiopia
Source: PLoS One. 2026 Aug 28;21(8):e0356876. doi: 10.1371/journal.pone.0356876 (PMC13524280; doi:10.1371/journal.pone.0356876)
Supplement: Supplementary file 3 [file pone.0356876.s003.docx]

S3

| AMF species | Count | RA (%) | IF (%) | Status |
| --- | --- | --- | --- | --- |
| *Acaulospora myriocarpa* | 11 | 78.57% | 78.57% | Dominant |
| *Glomus* sp | 4 | 28.57% | 28.57% | Common |
| *Pacispora* sp | 2 | 14.28% | 14.28% | Common |
| *Acaulospora delicate* | 1 | 7.14% | 7.14% | Rare |
| *Pacispora franciscana* | 10 | 71.43% | 71.43% | Dominant |
| *Acaulospora scrobiculata* | 2 | 14.28% | 14.28% | Common |
| *Acaulospora mellea* | 2 | 14.28% | 14.28% | Common |
| *Pacispora robiginia* | 1 | 7.14% | 7.14% | Rare |
| *Acaulospora* sp | 2 | 14.28% | 14.28% | Common |
