# Supplementary material for: Cropland type shapes arbuscular mycorrhizal fungi (AMF) population density and species diversity in Northwest Ethiopia
Source: PLoS One. 2026 Aug 28;21(8):e0356876. doi: 10.1371/journal.pone.0356876 (PMC13524280; doi:10.1371/journal.pone.0356876)
Supplement: Supplementary file 4 [file pone.0356876.s004.docx]

**Supplementary information 1**: farm and soil fertility management practices survey information collection tools from Teff and maize croplands at Aneded, Awabel and Gozamin districts

**DMU, College of NCS, Dept of Biology, farm and Soil fertility management practices, Data collection Tools from Aneded, Awabel and Gozamin Districts, East Gojam Zone**

**Soil samples collection (Teff and maize croplands)**

1. Previous cropping history: What crop was growing last year on that particular field?

**Fertilizer and pesticide application**

1. Urea----------------------------------------------------------------------------------------yes/no
2. DAP-----------------------------------------------------------------------------------------yes/no
3. Compost-----------------------------------------------------------------------------------yes/no
4. Manure===============================================yes/no
5. Pesticide application--------------------------------------------------------------------- yes/no
6. Lime application---------------------------------------------------------------------------yes/no

**Agricultural practice**

1. Line sowing------------------------------------------------------------------------------------
2. Intercropping---------------------------------------------------------------------------------
3. Traditional sowing----------------------------------------------------------------------------
4. Mention if any---------------------------------------------------------------------------------

**Season when the crop was sown**

1. Irrigation--------------------------------------------------------------------------------
2. Rain feed-------------------------------------------------------------------------------
3. Mention if any_________________________________________

**Plant/Seeds (wheat, teff)**

Seed varieties

1. ------------------ 2. ------------------ 3. ----------------- 4. ---------------------

**VI. Soil type/classes**

1. Black soil -----------------------------------------------------------------------------------
2. Red soil -----------------------------------------------------------------------------------
