# Supplementary material for: Cropland type shapes arbuscular mycorrhizal fungi (AMF) population density and species diversity in Northwest Ethiopia
Source: PLoS One. 2026 Aug 28;21(8):e0356876. doi: 10.1371/journal.pone.0356876 (PMC13524280; doi:10.1371/journal.pone.0356876)
Supplement: Supplementary file 5 [file pone.0356876.s006.docx]

**10.07.2026**

Point by point response to the reviewers

Cropland type shapes arbuscular mycorrhizal fungi (AMF) population density and species diversity in Northwest Ethiopia

PONE-D-26-14820

Dear	Dr. Ying Ma, Academic Editor and

Dr. Tancredo Souza and Prof. Joseph George Ray (Reviewers)

Many thanks for your contribution in our career development and giving this golden opportunity to revise and improve our manuscript. We highly acknowledge and appreciate your critical reviews, inputs and time invested to improve the quality of our manuscript. We learned a lot from your review comments and suggestions. We accepted all your general and specific comments and suggestion with full recognition. We have also made revision to the manuscript as per your comments and suggestions. Here below, we forward the point by point response for reviewers’ comments and suggestions.

With due respect

Belay Berza Beyene (the submitting author)

**July 10.2026**

**Point by point response to the reviewers**

**Reviewer #1**

**Comment 1**;  The manuscript entitled “Cropland type shapes arbuscular mycorrhizal fungi (AMF) population density and species diversity in Northwest Ethiopia” presents relevant and timely research addressing the interactions between agricultural management and AMF communities.

**Response to reviewer**; many thanks for your encouraging and positive view.

**Comment 2**: In the abstract (lines 11–28), the objectives and main findings are clearly stated; however, the opening sentence (lines 11–12) requires grammatical correction and improved clarity regarding the functional role of AMF. The phrase “absorption and translocation immobile plant nutrients” should be revised for readability. Additionally, the expression of results (lines 18–23) would benefit from more concise wording and consistent units formatting (e.g., “spores per 100 g dry soil”). The concluding sentence (lines 26–28) is somewhat generic and could be strengthened to better reflect the study’s specific contributions.

**Response to reviewer: We highly appreciate your comments and suggestions. All your comments and suggestions are incorporated accordingly in the abstract section.( lines 12-29)**

**Comment 3**: In the introduction (lines 34–74), the background is comprehensive and scientifically sound, but several sentences require linguistic polishing and reduction of redundancy. For example, lines 39–47 contain repetitive statements regarding nutrient uptake and AMF benefits. The hypotheses (lines 68–70) are appropriate, although their phrasing could be more precise (e.g., “resulting reduced population density” should be corrected). Additionally, the transition between general background and the study rationale (lines 62–67) could be made more concise and explicit.

**Response to reviewer: Many thanks for your comments and recommendations. We have incorporated all your comments in introduction section (lines 35-75)**
**Comment 4:** In the materials and methods section (lines 75–172), the methodology is generally well described and reproducible. However, some clarifications are needed. In the study area description (lines 76–84), minor grammatical issues and unit standardization (e.g., “300 kms”) should be corrected. In the sampling design (lines 91–104), the rationale for the sample size (14 croplands) should be briefly justified. The description of AMF extraction and identification (lines 116–150) is detailed, but some sentences are overly long and could be simplified for clarity. In the diversity indices subsection (lines 151–166), the formulas are not consistently presented (e.g., duplicated “H' = H' =”) and should be corrected. Additionally, the Simpson index formula appears incomplete or incorrectly formatted (line 158). The statistical analysis (lines 168–172) would benefit from specifying assumptions checking (e.g., normality, homoscedasticity).

**Response to reviewers:**

**Many thanks for your comments and suggestions. We have incorporated all your comments and concerns in the materials and method section (lines 77-181)**

**Comments 5:** In the results (lines 173–313), the findings are clearly presented and supported by tables and figures. However, several grammatical inconsistencies and typographical errors are present throughout (e.g., lines 190, 203–205, 257–259). The narrative occasionally repeats numerical values already shown in tables (e.g., lines 212–214 and corresponding tables), which could be streamlined. Some comparisons (e.g., lines 245–247) would benefit from clearer statistical interpretation rather than descriptive statements alone.

**Comment 6**: In the discussion (lines 314–396), the authors provide an adequate interpretation of the results in light of existing literature. Nonetheless, several sentences require grammatical revision (e.g., line 316 “playvital significance”) and improved clarity. Some arguments are repetitive, particularly regarding the effects of agricultural practices on AMF (lines 320–338). The discussion could be strengthened by more explicitly linking results to the stated hypotheses (lines 68–70). Additionally, certain statements would benefit from more cautious wording to avoid overgeneralization.

**Response to reviewers**: Many thanks for your comments and recommendations. We have incorporated all your comments and suggestions to improve the discussion (Lines 335-438).

**Comment 7**: In the conclusion (lines 397–404), the main findings are summarized appropriately. However, the phrasing (lines 397–401) should be revised for grammatical correctness and conciseness. The recommendation sentence (lines 401–404) could be made more specific by indicating which practices are most relevant based on the results.

**Response to reviewer:** Many thanks for your comments and recommendations. We fully accepted your comments with respect and incorporated all your comments to enhance our conclusions (lines 440-446).

**Comment 8**: Regarding the tables (lines 182–299), they are informative but require formatting improvements. Several table titles lack proper capitalization and clarity (e.g., “Table 1 the preliminary survey results…”). Units and variable descriptions should be standardized across all tables. Some typographical inconsistencies are present (e.g., species names formatting in Tables 7 and 8), and scientific names should be consistently italicized.

**Response to reviewers**: done

**Comment 9**: The figures (lines 248–262) are relevant to the results, but their captions should be more descriptive and self-explanatory. Axis labels require standardization (e.g., “gram dry soil” vs. “g dry soil”), and grammatical corrections are needed. The statistical groupings (letters a, b, c) should be clearly explained in the captions.

**Response to reviewers**: done

**Comments 10**: As additional comments, the manuscript would benefit from a thorough language revision by a fluent English speaker to address recurrent grammatical and stylistic issues. Consistency in terminology (e.g., “spore density,” “population density”) should be ensured throughout. The data availability statement (lines 413–414) should be aligned with PLOS ONE policy, as “data available on request” is typically not sufficient; the authors should clarify whether data are included in the manuscript or deposited in a repository. Finally, minor formatting inconsistencies in references and in-text citations should be corrected.

**Response to reviewers**: done

**Reviewer #2:**

**Comment 1**: This article presents a well-written, systematic study on the impact of different crops and soil characteristics on AMF population density and species diversity in Ethiopia. The introduction effectively explains the research background, problem origin, hypothesis, and objectives. However, some of the recent reports on ecology of mycorrhiza in crop fields (e.g.: <https://doi.org/10.3389/fsufs.2023.1124688>; <https://doi.org/10.1016/j.jarmap.2023.100507> ; <https://doi.org/10.1093/jambio/lxad111> ; <https://journal.univ-eloued.dz/index.php/ajb/login?source=%2Findex.php%2Fajb%2Farticle%2Fdownload%2F179%2F148%2F224> ) can be referred to improve the logic.

**Response to reviewers: Many thanks for your comments and suggestions. Comments are incorporated and done**

**Comment 2**: The methodology is well-explained, ensuring reproducibility, and the findings are significant. However, the interpretations in the discussion are not up-to-date with recent findings regarding mycorrhizal ecology (e.g.: <https://doi.org/10.1016/j.geodrs.2025.e00992>; <https://doi.org/10.1016/j.jarmap.2024.100617>; <https://doi.org/10.1093/jambio/lxae256> ; <https://doi.org/10.1016/j.rhisph.2024.100961> ). They can use these and other similar references on the ecology of mycorrhiza in crop fields to improve the discussion. However, the authors should consult recent articles and thoroughly revise the discussion. The conclusion is precise but should be revised based on the updated discussion. Furthermore, numerous typos require thorough correction. I recommend publication pending a thorough revision of the discussion and correction of typos throughout the manuscript. Note that the suggested typos are examples, and a comprehensive check is necessary.

**Response to reviewers: Many thanks for your comments and suggestions. Comments are incorporated and done**

**Comment 3:** Examples of Typos observed, several listed.
**Response: Many thanks for your careful and critical review. We have accepted all your comments and incorporated all your comments and done.**
