# Supplementary material for: Cropland type shapes arbuscular mycorrhizal fungi (AMF) population density and species diversity in Northwest Ethiopia
Source: PLoS One. 2026 Aug 28;21(8):e0356876. doi: 10.1371/journal.pone.0356876 (PMC13524280; doi:10.1371/journal.pone.0356876)
Supplement: Supplementary file 6 [file pone.0356876.s007.docx]

**05.08.2026**

Point by point response to the reviewers

Cropland type shapes arbuscular mycorrhizal fungi (AMF) population density and species diversity in Northwest Ethiopia

PONE-D-26-14820R1

Dear	Dr. Ying Ma, Academic Editor and

Dr. Tancredo Souza and Prof. Joseph George Ray (Reviewers)

Many thanks for your contribution in our career development and for the second time careful review to improve our manuscript. We highly acknowledge and appreciate your critical reviews, inputs and time invested to improve the quality of our manuscript. We still learned a lot from your review comments and suggestions. We accepted all your general and specific comments and suggestion with full recognition. We have also made revision to the manuscript as per your comments and suggestions. Here below, we forward the point by point response for reviewers’ comments and suggestions.

With due respect

Belay Berza Beyene (the submitting author)

**05.08.2026**

**Point by point response to reviewers**

**Reviewer #1**

**Comment 1**; As Figures 1-3 illustrate the impact of various factors on field spore densities, Tables 1-4 can be moved to a supplementary appendix.

**Response by authors: Thanks for your comments. We accept and took Table 1 to appendix as supplementary table. However, Tables 2-4 are left in place because they exhibit specific sampling locations while figs show at district level. That means they are not the same. I hope you understand my point. Thanks**

**Comment 2**. The unnumbered table above Figure 4 is unnecessary.

**Response by authors: thanks again. The unnamed table was Table 6. We took it to the appendix as supplementary.**

**Comment 3**. Table 8 includes repetition of data in Table 7; both may be combined into a single table.

**Response by authors: thanks again. We accept your comments. We took table 8 to appendix. However, we created Fig.5 from table 8.**

**Comment 4**. The details of Table 9 can be mentioned in one of two lines in the text, and therefore, Table 9 can be avoided.

**Response by authors: Thanks again. We believe that table explain things more than words. |Therefore, we kept it there.**

**Comment 5**. References 11-14 - please note that these references in lines 58-60 are pretty old – many recent studies in the same context are available in recent literature

**Response by authors: Many thanks. We accept your comments and it is done as per your comments**

**Comment 6**. Reference 24 – The citation No. 24 in Line 336 is pretty old; the same findings are available in recent literature

**Response by authors: Many thanks. We accept your comments and it is done as per your comments**

**Comment 7**. Reference 26 – in line 343 is pretty old; better confirmations of a similar nature are available in recent literature, which needs to be cited here

**Response by authors: Many thanks. We accept your comments and it is done as per your comments**

**Comments 8**. References 31, 33-34, 38, 42-44 are pretty old references, which need to be replaced by recent, up-to-date citations with better confirmations in these regards.

**Response by authors: Many thanks. We accept your comments and it is done as per your comments**
