# Supplementary material for: Cropland type shapes arbuscular mycorrhizal fungi (AMF) population density and species diversity in Northwest Ethiopia
Source: PLoS One. 2026 Aug 28;21(8):e0356876. doi: 10.1371/journal.pone.0356876 (PMC13524280; doi:10.1371/journal.pone.0356876)
Supplement: Peer review history

# Peer review: Decision Letter 0

**DOI:** 10.1371/journal.pone.0356876.r001

**Authors:** Ying Ma

2 Jun 2026

Dear Dr. Beyene,

Thank you for submitting your manuscript to PLOS ONE. After careful consideration, we feel that it has merit but does not fully meet PLOS ONE’s publication criteria as it currently stands. Therefore, we invite you to submit a revised version of the manuscript that addresses the points raised during the review process.

Please submit your revised manuscript by Jul 17 2026 11:59PM. If you will need more time than this to complete your revisions, please reply to this message or contact the journal office at plosone@plos.org. When you're ready to submit your revision, log on to https://www.editorialmanager.com/pone/ and select the 'Submissions Needing Revision' folder to locate your manuscript file.

- A letter that responds to each point raised by the academic editor and reviewer(s). You should upload this letter as a separate file labeled 'Response to Reviewers'.
- A marked-up copy of your manuscript that highlights changes made to the original version. You should upload this as a separate file labeled 'Revised Manuscript with Track Changes'.
- An unmarked version of your revised paper without tracked changes. You should upload this as a separate file labeled 'Manuscript'.

If you would like to make changes to your financial disclosure, please include your updated statement in your cover letter. Guidelines for resubmitting your figure files are available below the reviewer comments at the end of this letter.

If applicable, we recommend that you deposit your laboratory protocols in protocols.io to enhance the reproducibility of your results. Protocols.io assigns your protocol its own identifier (DOI) so that it can be cited independently in the future. For instructions see: https://journals.plos.org/plosone/s/submission-guidelines#loc-laboratory-protocols. Additionally, PLOS ONE offers an option for publishing peer-reviewed Lab Protocol articles, which describe protocols hosted on protocols.io. Read more information on sharing protocols at https://plos.org/protocols?utm_medium=editorial-email&utm_source=authorletters&utm_campaign=protocols.

As the corresponding author, your ORCID iD is verified in the submission system and will appear in the published article. PLOS supports the use of ORCID, and we encourage all coauthors to register for an ORCID iD and use it as well. Please encourage your coauthors to verify their ORCID iD within the submission system before final acceptance, as unverified ORCID iDs will not appear in the published article. *Only*  the individual author can complete the verification step; PLOS staff *cannot*  verify ORCID iDs on behalf of authors.

We look forward to receiving your revised manuscript.

Kind regards,

Ying Ma, Ph.D.

Academic Editor

PLOS One

Journal Requirements:

When submitting your revision, we need you to address these additional requirements.

1. Please ensure that your manuscript meets PLOS ONE's style requirements, including those for file naming. The PLOS ONE style templates can be found at

https://journals.plos.org/plosone/s/file?id=wjVg/PLOSOne_formatting_sample_main_body.pdf and https://journals.plos.org/plosone/s/file?id=ba62/PLOSOne_formatting_sample_title_authors_affiliations.pdf

2. Please provide additional details regarding participant consent for survey. In the Methods and online submission information, please ensure that you have specified (1) whether consent was informed and (2) what type was obtained (for instance, written or verbal, and if verbal, how it was documented and witnessed).

3. In your Methods section, please provide additional information regarding the permits you obtained for the work. Please ensure you have included the full name of the authority that approved the field site access and, if no permits were required, a brief statement explaining why.

4. Please provide a complete Data Availability Statement in the submission form, ensuring you include all necessary access information or a reason for why you are unable to make your data freely accessible. If your research concerns only data provided within your submission, please write "All data are in the manuscript and/or supporting information files" as your Data Availability Statement.

5. Please amend your authorship list in your manuscript file to include author Belay Berza Beyene.

6. Please amend the manuscript submission data (via Edit Submission) to include author Belay Berza.

7. Please include captions for your Supporting Information files at the end of your manuscript, and update any in-text citations to match accordingly. Please see our Supporting Information guidelines for more information: http://journals.plos.org/plosone/s/supporting-information.

8. We note that Figure S1 in your submission contain copyrighted images. All PLOS content is published under the Creative Commons Attribution License (CC BY 4.0), which means that the manuscript, images, and Supporting Information files will be freely available online, and any third party is permitted to access, download, copy, distribute, and use these materials in any way, even commercially, with proper attribution. For more information, see our copyright guidelines: http://journals.plos.org/plosone/s/licenses-and-copyright.

We require you to either (1) present written permission from the copyright holder to publish these figures specifically under the CC BY 4.0 license, or (2) remove the figures from your submission:

a. You may seek permission from the original copyright holder of Figure S1 to publish the content specifically under the CC BY 4.0 license.

We recommend that you contact the original copyright holder with the Content Permission Form (http://journals.plos.org/plosone/s/file?id=7c09/content-permission-form.pdf) and the following text:

“I request permission for the open-access journal PLOS ONE to publish XXX under the Creative Commons Attribution License (CCAL) CC BY 4.0 (http://creativecommons.org/licenses/by/4.0/). Please be aware that this license allows unrestricted use and distribution, even commercially, by third parties. Please reply and provide explicit written permission to publish XXX under a CC BY license and complete the attached form.”

Please upload the completed Content Permission Form or other proof of granted permissions as an "Other" file with your submission.

In the figure caption of the copyrighted figure, please include the following text: “Reprinted from [ref] under a CC BY license, with permission from [name of publisher], original copyright [original copyright year].”

b. If you are unable to obtain permission from the original copyright holder to publish these figures under the CC BY 4.0 license or if the copyright holder’s requirements are incompatible with the CC BY 4.0 license, please either i) remove the figure or ii) supply a replacement figure that complies with the CC BY 4.0 license. Please check copyright information on all replacement figures and update the figure caption with source information. If applicable, please specify in the figure caption text when a figure is similar but not identical to the original image and is therefore for illustrative purposes only.

9. If the reviewer comments include a recommendation to cite specific previously published works, please review and evaluate these publications to determine whether they are relevant and should be cited. There is no requirement to cite these works unless the editor has indicated otherwise.

[Note: HTML markup is below. Please do not edit.]

Reviewers' comments:

Reviewer's Responses to Questions

**Comments to the Author**

1. Is the manuscript technically sound, and do the data support the conclusions?

Reviewer #1: Partly

Reviewer #2: Yes

**********

2. Has the statistical analysis been performed appropriately and rigorously?

Reviewer #1: Yes

Reviewer #2: Yes

**********

3. Have the authors made all data underlying the findings in their manuscript fully available?

The PLOS Data policy

Reviewer #1: Yes

Reviewer #2: Yes

**********

4. Is the manuscript presented in an intelligible fashion and written in standard English?

Reviewer #1: Yes

Reviewer #2: Yes

**********

Reviewer #1: The manuscript entitled “Cropland type shapes arbuscular mycorrhizal fungi (AMF) population density and species diversity in Northwest Ethiopia” presents relevant and timely research addressing the interactions between agricultural management and AMF communities. The study is within the scope of PLOS ONE and contributes useful data from an underrepresented geographic region. Overall, the manuscript is well-structured and the results are of potential interest; however, several aspects require clarification and linguistic refinement before publication. I recommend acceptance after minor revision.

In the abstract (lines 11–28), the objectives and main findings are clearly stated; however, the opening sentence (lines 11–12) requires grammatical correction and improved clarity regarding the functional role of AMF. The phrase “absorption and translocation immobile plant nutrients” should be revised for readability. Additionally, the expression of results (lines 18–23) would benefit from more concise wording and consistent units formatting (e.g., “spores per 100 g dry soil”). The concluding sentence (lines 26–28) is somewhat generic and could be strengthened to better reflect the study’s specific contributions.

In the introduction (lines 34–74), the background is comprehensive and scientifically sound, but several sentences require linguistic polishing and reduction of redundancy. For example, lines 39–47 contain repetitive statements regarding nutrient uptake and AMF benefits. The hypotheses (lines 68–70) are appropriate, although their phrasing could be more precise (e.g., “resulting reduced population density” should be corrected). Additionally, the transition between general background and the study rationale (lines 62–67) could be made more concise and explicit.

In the materials and methods section (lines 75–172), the methodology is generally well described and reproducible. However, some clarifications are needed. In the study area description (lines 76–84), minor grammatical issues and unit standardization (e.g., “300 kms”) should be corrected. In the sampling design (lines 91–104), the rationale for the sample size (14 croplands) should be briefly justified. The description of AMF extraction and identification (lines 116–150) is detailed, but some sentences are overly long and could be simplified for clarity. In the diversity indices subsection (lines 151–166), the formulas are not consistently presented (e.g., duplicated “H' = H' =”) and should be corrected. Additionally, the Simpson index formula appears incomplete or incorrectly formatted (line 158). The statistical analysis (lines 168–172) would benefit from specifying assumptions checking (e.g., normality, homoscedasticity).

In the results (lines 173–313), the findings are clearly presented and supported by tables and figures. However, several grammatical inconsistencies and typographical errors are present throughout (e.g., lines 190, 203–205, 257–259). The narrative occasionally repeats numerical values already shown in tables (e.g., lines 212–214 and corresponding tables), which could be streamlined. Some comparisons (e.g., lines 245–247) would benefit from clearer statistical interpretation rather than descriptive statements alone.

In the discussion (lines 314–396), the authors provide an adequate interpretation of the results in light of existing literature. Nonetheless, several sentences require grammatical revision (e.g., line 316 “playvital significance”) and improved clarity. Some arguments are repetitive, particularly regarding the effects of agricultural practices on AMF (lines 320–338). The discussion could be strengthened by more explicitly linking results to the stated hypotheses (lines 68–70). Additionally, certain statements would benefit from more cautious wording to avoid overgeneralization.

In the conclusion (lines 397–404), the main findings are summarized appropriately. However, the phrasing (lines 397–401) should be revised for grammatical correctness and conciseness. The recommendation sentence (lines 401–404) could be made more specific by indicating which practices are most relevant based on the results.

Regarding the tables (lines 182–299), they are informative but require formatting improvements. Several table titles lack proper capitalization and clarity (e.g., “Table 1 the preliminary survey results…”). Units and variable descriptions should be standardized across all tables. Some typographical inconsistencies are present (e.g., species names formatting in Tables 7 and 8), and scientific names should be consistently italicized.

The figures (lines 248–262) are relevant to the results, but their captions should be more descriptive and self-explanatory. Axis labels require standardization (e.g., “gram dry soil” vs. “g dry soil”), and grammatical corrections are needed. The statistical groupings (letters a, b, c) should be clearly explained in the captions.

As additional comments, the manuscript would benefit from a thorough language revision by a fluent English speaker to address recurrent grammatical and stylistic issues. Consistency in terminology (e.g., “spore density,” “population density”) should be ensured throughout. The data availability statement (lines 413–414) should be aligned with PLOS ONE policy, as “data available on request” is typically not sufficient; the authors should clarify whether data are included in the manuscript or deposited in a repository. Finally, minor formatting inconsistencies in references and in-text citations should be corrected.

Reviewer #2: This article presents a well-written, systematic study on the impact of different crops and soil characteristics on AMF population density and species diversity in Ethiopia. The introduction effectively explains the research background, problem origin, hypothesis, and objectives. However, some of the recent reports on ecology of mycorrhiza in crop fields (e.g.: https://doi.org/10.3389/fsufs.2023.1124688; https://doi.org/10.1016/j.jarmap.2023.100507 ; https://doi.org/10.1093/jambio/lxad111 ; https://journal.univ-eloued.dz/index.php/ajb/login?source=%2Findex.php%2Fajb%2Farticle%2Fdownload%2F179%2F148%2F224 ) can be referred to improve the logic.

The methodology is well-explained, ensuring reproducibility, and the findings are significant. However, the interpretations in the discussion are not up-to-date with recent findings regarding mycorrhizal ecology (e.g.: https://doi.org/10.1016/j.geodrs.2025.e00992; https://doi.org/10.1016/j.jarmap.2024.100617; https://doi.org/10.1093/jambio/lxae256 ; https://doi.org/10.1016/j.rhisph.2024.100961 ). They can use these and other similar references on the ecology of mycorrhiza in crop fields to improve the discussion.

However, the authors should consult recent articles and thoroughly revise the discussion. The conclusion is precise but should be revised based on the updated discussion.

Furthermore, numerous typos require thorough correction. I recommend publication pending a thorough revision of the discussion and correction of typos throughout the manuscript. Note that the suggested typos are examples, and a comprehensive check is necessary.

Examples of Typos observed

Line 12: ‘translocation immobile’ – translocation of immobile

Line 76: ‘2.1. Description of the research Area’ – this subheading may be modified as ‘2.1. Area of study.

Line 93: ‘are selected’ – were selected

Line 94: ‘A total of 14 croplands’ – tell the size of each cropland, ranging from the smallest to the largest size

Line 99: ‘a sterile polythene bags’ – not a bags, but in sterile polythene bags

Line 108: ‘Prior to analysis’ – prior to soil chemical analysis

Line 124: ‘any found’ – spores found

Line 138: ‘by the Glomeromycota database’ – cite the URL of the site in brackets

Line 175: ‘black soils’ – mention the USDA soil type (soil order and soil series)

Line 176: ‘Red soils’ - mention the USDA soil type (soil order and soil series)

Table 1: Column 8: give the rate of fertilization application practiced in the region per hectare

Line 187: ‘intensive chemical fertilizer and pesticide applications’ – confirm the rate of fertilizer and pesticide applications in the region.

Line 190: ‘varied across the at the’ – correct the sentence structure

Line 316: ‘playvital’ – play vital

**********

what does this mean?). If published, this will include your full peer review and any attached files.

If you choose “no”, your identity will remain anonymous but your review may still be made public.

**Do you want your identity to be public for this peer review?**  For information about this choice, including consent withdrawal, please see our Privacy Policy

Reviewer #1: **Yes:** Dr. Tancredo Souza

Reviewer #2: **Yes:** Prof Joseph George Ray, School of Biosciences, Mahatma Gandhi University, Kottayam, Kerala

**********

[NOTE: If reviewer comments were submitted as an attachment file, they will be attached to this email and accessible via the submission site. Please log into your account, locate the manuscript record, and check for the action link "View Attachments". If this link does not appear, there are no attachment files.]

To ensure your figures meet our technical requirements, please review our figure guidelines: https://journals.plos.org/plosone/s/figures

You may also use PLOS’s free figure tool, NAAS, to help you prepare publication quality figures: https://journals.plos.org/plosone/s/figures#loc-tools-for-figure-preparation.

NAAS will assess whether your figures meet our technical requirements by comparing each figure against our figure specifications.

# Author comment: Author response to Decision Letter 1

**DOI:** 10.1371/journal.pone.0356876.r002

11 Jul 2026

10.07.2026

Point by point response to the reviewers

Cropland type shapes arbuscular mycorrhizal fungi (AMF) population density and species diversity in Northwest Ethiopia

PONE-D-26-14820

Dear Dr. Ying Ma, Academic Editor and

Dr. Tancredo Souza and Prof. Joseph George Ray (Reviewers)

Many thanks for your contribution in our career development and giving this golden opportunity to revise and improve our manuscript. We highly acknowledge and appreciate your critical reviews, inputs and time invested to improve the quality of our manuscript. We learned a lot from your review comments and suggestions. We accepted all your general and specific comments and suggestion with full recognition. We have also made revision to the manuscript as per your comments and suggestions. Here below, we forward the point by point response for reviewers’ comments and suggestions.

With due respect

Belay Berza Beyene (the submitting author)

July 10.2026

Point by point response to the reviewers

Reviewer #1

Comment 1; The manuscript entitled “Cropland type shapes arbuscular mycorrhizal fungi (AMF) population density and species diversity in Northwest Ethiopia” presents relevant and timely research addressing the interactions between agricultural management and AMF communities.

Response to reviewer; many thanks for your encouraging and positive view.

Comment 2: In the abstract (lines 11–28), the objectives and main findings are clearly stated; however, the opening sentence (lines 11–12) requires grammatical correction and improved clarity regarding the functional role of AMF. The phrase “absorption and translocation immobile plant nutrients” should be revised for readability. Additionally, the expression of results (lines 18–23) would benefit from more concise wording and consistent units formatting (e.g., “spores per 100 g dry soil”). The concluding sentence (lines 26–28) is somewhat generic and could be strengthened to better reflect the study’s specific contributions.

Response to reviewer: We highly appreciate your comments and suggestions. All your comments and suggestions are incorporated accordingly in the abstract section.( lines 12-29)

Comment 3: In the introduction (lines 34–74), the background is comprehensive and scientifically sound, but several sentences require linguistic polishing and reduction of redundancy. For example, lines 39–47 contain repetitive statements regarding nutrient uptake and AMF benefits. The hypotheses (lines 68–70) are appropriate, although their phrasing could be more precise (e.g., “resulting reduced population density” should be corrected). Additionally, the transition between general background and the study rationale (lines 62–67) could be made more concise and explicit.

Response to reviewer: Many thanks for your comments and recommendations. We have incorporated all your comments in introduction section (lines 35-75)

Comment 4: In the materials and methods section (lines 75–172), the methodology is generally well described and reproducible. However, some clarifications are needed. In the study area description (lines 76–84), minor grammatical issues and unit standardization (e.g., “300 kms”) should be corrected. In the sampling design (lines 91–104), the rationale for the sample size (14 croplands) should be briefly justified. The description of AMF extraction and identification (lines 116–150) is detailed, but some sentences are overly long and could be simplified for clarity. In the diversity indices subsection (lines 151–166), the formulas are not consistently presented (e.g., duplicated “H' = H' =”) and should be corrected. Additionally, the Simpson index formula appears incomplete or incorrectly formatted (line 158). The statistical analysis (lines 168–172) would benefit from specifying assumptions checking (e.g., normality, homoscedasticity).

Response to reviewers:

Many thanks for your comments and suggestions. We have incorporated all your comments and concerns in the materials and method section (lines 77-181)

Comments 5: In the results (lines 173–313), the findings are clearly presented and supported by tables and figures. However, several grammatical inconsistencies and typographical errors are present throughout (e.g., lines 190, 203–205, 257–259). The narrative occasionally repeats numerical values already shown in tables (e.g., lines 212–214 and corresponding tables), which could be streamlined. Some comparisons (e.g., lines 245–247) would benefit from clearer statistical interpretation rather than descriptive statements alone.

Comment 6: In the discussion (lines 314–396), the authors provide an adequate interpretation of the results in light of existing literature. Nonetheless, several sentences require grammatical revision (e.g., line 316 “playvital significance”) and improved clarity. Some arguments are repetitive, particularly regarding the effects of agricultural practices on AMF (lines 320–338). The discussion could be strengthened by more explicitly linking results to the stated hypotheses (lines 68–70). Additionally, certain statements would benefit from more cautious wording to avoid overgeneralization.

Response to reviewers: Many thanks for your comments and recommendations. We have incorporated all your comments and suggestions to improve the discussion (Lines 335-438).

Comment 7: In the conclusion (lines 397–404), the main findings are summarized appropriately. However, the phrasing (lines 397–401) should be revised for grammatical correctness and conciseness. The recommendation sentence (lines 401–404) could be made more specific by indicating which practices are most relevant based on the results.

Response to reviewer: Many thanks for your comments and recommendations. We fully accepted your comments with respect and incorporated all your comments to enhance our conclusions (lines 440-446).

Comment 8: Regarding the tables (lines 182–299), they are informative but require formatting improvements. Several table titles lack proper capitalization and clarity (e.g., “Table 1 the preliminary survey results…”). Units and variable descriptions should be standardized across all tables. Some typographical inconsistencies are present (e.g., species names formatting in Tables 7 and 8), and scientific names should be consistently italicized.

Response to reviewers: done

Comment 9: The figures (lines 248–262) are relevant to the results, but their captions should be more descriptive and self-explanatory. Axis labels require standardization (e.g., “gram dry soil” vs. “g dry soil”), and grammatical corrections are needed. The statistical groupings (letters a, b, c) should be clearly explained in the captions.

Response to reviewers: done

Comments 10: As additional comments, the manuscript would benefit from a thorough language revision by a fluent English speaker to address recurrent grammatical and stylistic issues. Consistency in terminology (e.g., “spore density,” “population density”) should be ensured throughout. The data availability statement (lines 413–414) should be aligned with PLOS ONE policy, as “data available on request” is typically not sufficient; the authors should clarify whether data are included in the manuscript or deposited in a repository. Finally, minor formatting inconsistencies in references and in-text citations should be corrected.

Response to reviewers: done

Reviewer #2:

Comment 1: This article presents a well-written, systematic study on the impact of different crops and soil characteristics on AMF population density and species diversity in Ethiopia. The introduction effectively explains the research background, problem origin, hypothesis, and objectives. However, some of the recent reports on ecology of mycorrhiza in crop fields (e.g.: https://doi.org/10.3389/fsufs.2023.1124688; https://doi.org/10.1016/j.jarmap.2023.100507 ; https://doi.org/10.1093/jambio/lxad111 ; https://journal.univ-eloued.dz/index.php/ajb/login?source=%2Findex.php%2Fajb%2Farticle%2Fdownload%2F179%2F148%2F224 ) can be referred to improve the logic.

Response to reviewers: Many thanks for your comments and suggestions. Comments are incorporated and done

Comment 2: The methodology is well-explained, ensuring reproducibility, and the findings are significant. However, the interpretations in the discussion are not up-to-date with recent findings regarding mycorrhizal ecology (e.g.: https://doi.org/10.1016/j.geodrs.2025.e00992; https://doi.org/10.1016/j.jarmap.2024.100617; https://doi.org/10.1093/jambio/lxae256 ; https://doi.org/10.1016/j.rhisph.2024.100961 ). They can use these and other similar references on the ecology of mycorrhiza in crop fields to improve the discussion. However, the authors should consult recent articles and thoroughly revise the discussion. The conclusion is precise but should be revised based on the updated discussion. Furthermore, numerous typos require thorough correction. I recommend publication pending a thorough revision of the discussion and correction of typos throughout the manuscript. Note that the suggested typos are examples, and a comprehensive check is necessary.

Response to reviewers: Many thanks for your comments and suggestions. Comments are incorporated and done

Comment 3: Examples of Typos observed, several listed.

Response: Many thanks for your careful and critical review. We have accepted all your comments and incorporated all your comments and done.

Attachment Submitted filename: Response to Reviewers.docx

# Peer review: Decision Letter 1

**DOI:** 10.1371/journal.pone.0356876.r003

**Authors:** Ying Ma

22 Jul 2026

Dear Dr. Beyene,

Thank you for submitting your manuscript to PLOS ONE. After careful consideration, we feel that it has merit but does not fully meet PLOS ONE’s publication criteria as it currently stands. Therefore, we invite you to submit a revised version of the manuscript that addresses the points raised during the review process.

Please submit your revised manuscript by Sep 05 2026 11:59PM. If you will need more time than this to complete your revisions, please reply to this message or contact the journal office at plosone@plos.org. When you're ready to submit your revision, log on to https://www.editorialmanager.com/pone/ and select the 'Submissions Needing Revision' folder to locate your manuscript file.

- A letter that responds to each point raised by the academic editor and reviewer(s). You should upload this letter as a separate file labeled 'Response to Reviewers'.
- A marked-up copy of your manuscript that highlights changes made to the original version. You should upload this as a separate file labeled 'Revised Manuscript with Track Changes'.
- An unmarked version of your revised paper without tracked changes. You should upload this as a separate file labeled 'Manuscript'.

If you would like to make changes to your financial disclosure, please include your updated statement in your cover letter. Guidelines for resubmitting your figure files are available below the reviewer comments at the end of this letter.

If applicable, we recommend that you deposit your laboratory protocols in protocols.io to enhance the reproducibility of your results. Protocols.io assigns your protocol its own identifier (DOI) so that it can be cited independently in the future. For instructions see: https://journals.plos.org/plosone/s/submission-guidelines#loc-laboratory-protocols. Additionally, PLOS ONE offers an option for publishing peer-reviewed Lab Protocol articles, which describe protocols hosted on protocols.io. Read more information on sharing protocols at https://plos.org/protocols?utm_medium=editorial-email&utm_source=authorletters&utm_campaign=protocols.

As the corresponding author, your ORCID iD is verified in the submission system and will appear in the published article. PLOS supports the use of ORCID, and we encourage all coauthors to register for an ORCID iD and use it as well. Please encourage your coauthors to verify their ORCID iD within the submission system before final acceptance, as unverified ORCID iDs will not appear in the published article. *Only*  the individual author can complete the verification step; PLOS staff *cannot*  verify ORCID iDs on behalf of authors.

We look forward to receiving your revised manuscript.

Kind regards,

Ying Ma, Ph.D.

Academic Editor

PLOS One

Journal Requirements:

If the reviewer comments include a recommendation to cite specific previously published works, please review and evaluate these publications to determine whether they are relevant and should be cited. There is no requirement to cite these works unless the editor has indicated otherwise.

Please review your reference list to ensure that it is complete and correct. If you have cited papers that have been retracted, please include the rationale for doing so in the manuscript text, or remove these references and replace them with relevant current references. Any changes to the reference list should be mentioned in the rebuttal letter that accompanies your revised manuscript. If you need to cite a retracted article, indicate the article’s retracted status in the References list and also include a citation and full reference for the retraction notice.

[Note: HTML markup is below. Please do not edit.]

Reviewers' comments:

Reviewer's Responses to Questions

**Comments to the Author**

Reviewer #1: All comments have been addressed

Reviewer #2: (No Response)

**********

2. Is the manuscript technically sound, and do the data support the conclusions?

Reviewer #1: Yes

Reviewer #2: Partly

**********

3. Has the statistical analysis been performed appropriately and rigorously?

Reviewer #1: Yes

Reviewer #2: N/A

**********

4. Have the authors made all data underlying the findings in their manuscript fully available?

The PLOS Data policy

Reviewer #1: Yes

Reviewer #2: Yes

**********

5. Is the manuscript presented in an intelligible fashion and written in standard English?

Reviewer #1: Yes

Reviewer #2: No

**********

Reviewer #1: Based on my evaluation of the revised manuscript and the authors' detailed responses to the reviewers' comments, I conclude that the manuscript has been substantially improved. The authors have carefully addressed the comments raised during the review process by clarifying the experimental design, strengthening the Introduction and Discussion, improving the description of the methodology and statistical analyses, revising the tables and figures, acknowledging the study's limitations, and correcting numerous language and formatting issues. The point-by-point responses are comprehensive, thoughtful, and demonstrate the authors' genuine commitment to improving the scientific quality and clarity of their work.

The revised manuscript is now scientifically sound, well organized, and presents its findings in a clear and coherent manner. The conclusions are adequately supported by the data presented, and the study provides valuable information for the field.

Therefore, I recommend the manuscript for publication in its current form.

I congratulate the authors on their diligent revision and for the considerable improvements made to the manuscript. Their careful attention to the reviewers' comments has significantly strengthened the overall quality of the study.

Reviewer #2: The authors have significantly improved the presentation by incorporating feedback. However, further refinements are essential before the article is considered for publication. Although the authors answered that they have revised the text with recent references, many pretty old ones persist in the text. Moreover, many tables seem unnecessary in the main text.

Suggestions for improvement

1. As Figures 1-3 illustrate the impact of various factors on field spore densities, Tables 1-4 can be moved to a supplementary appendix.

2. The unnumbered table above Figure 4 is unnecessary.

3. Table 8 includes repetition of data in Table 7; both may be combined into a single table.

4. The details of Table 9 can be mentioned in one of two lines in the text, and therefore, Table 9 can be avoided.

5. References 11-14 - please note that these references in lines 58-60 are pretty old – many recent studies in the same context are available in recent literature

6. Reference 24 – The citation No. 24 in Line 336 is pretty old; the same findings are available in recent literature

7. Reference 26 – in line 343 is pretty old; better confirmations of a similar nature are available in recent literature, which needs to be cited here

8. References 31, 33-34, 38, 42-44 are pretty old references, which need to be replaced by recent, up-to-date citations with better confirmations in these regards.

**********

what does this mean?). If published, this will include your full peer review and any attached files.

If you choose “no”, your identity will remain anonymous but your review may still be made public.

**Do you want your identity to be public for this peer review?**  For information about this choice, including consent withdrawal, please see our Privacy Policy

Reviewer #1: **Yes:** Dr. Tancredo Souza

Reviewer #2: **Yes:** Joseph George Ray

**********

[NOTE: If reviewer comments were submitted as an attachment file, they will be attached to this email and accessible via the submission site. Please log into your account, locate the manuscript record, and check for the action link "View Attachments". If this link does not appear, there are no attachment files.]

To ensure your figures meet our technical requirements, please review our figure guidelines: https://journals.plos.org/plosone/s/figures

You may also use PLOS’s free figure tool, NAAS, to help you prepare publication quality figures: https://journals.plos.org/plosone/s/figures#loc-tools-for-figure-preparation.

NAAS will assess whether your figures meet our technical requirements by comparing each figure against our figure specifications.

# Author comment: Author response to Decision Letter 2

**DOI:** 10.1371/journal.pone.0356876.r004

7 Aug 2026

05.08.2026

Point by point response to the reviewers

Cropland type shapes arbuscular mycorrhizal fungi (AMF) population density and species diversity in Northwest Ethiopia

PONE-D-26-14820R1

Dear Dr. Ying Ma, Academic Editor and

Dr. Tancredo Souza and Prof. Joseph George Ray (Reviewers)

Many thanks for your contribution in our career development and for the second time careful review to improve our manuscript. We highly acknowledge and appreciate your critical reviews, inputs and time invested to improve the quality of our manuscript. We still learned a lot from your review comments and suggestions. We accepted all your general and specific comments and suggestion with full recognition. We have also made revision to the manuscript as per your comments and suggestions. Here below, we forward the point by point response for reviewers’ comments and suggestions.

With due respect

Belay Berza Beyene (the submitting author)

05.08.2026

Point by point response to reviewers

Reviewer #1

Comment 1; As Figures 1-3 illustrate the impact of various factors on field spore densities, Tables 1-4 can be moved to a supplementary appendix.

Response by authors: Thanks for your comments. We accept and took Table 1 to appendix as supplementary table. However, Tables 2-4 are left in place because they exhibit specific sampling locations while figs show at district level. That means they are not the same. I hope you understand my point. Thanks

Comment 2. The unnumbered table above Figure 4 is unnecessary.

Response by authors: thanks again. The unnamed table was Table 6. We took it to the appendix as supplementary.

Comment 3. Table 8 includes repetition of data in Table 7; both may be combined into a single table.

Response by authors: thanks again. We accept your comments. We took table 8 to appendix. However, we created Fig.5 from table 8.

Comment 4. The details of Table 9 can be mentioned in one of two lines in the text, and therefore, Table 9 can be avoided.

Response by authors: Thanks again. We believe that table explain things more than words. |Therefore, we kept it there.

Comment 5. References 11-14 - please note that these references in lines 58-60 are pretty old – many recent studies in the same context are available in recent literature

Response by authors: Many thanks. We accept your comments and it is done as per your comments

Comment 6. Reference 24 – The citation No. 24 in Line 336 is pretty old; the same findings are available in recent literature

Response by authors: Many thanks. We accept your comments and it is done as per your comments

Comment 7. Reference 26 – in line 343 is pretty old; better confirmations of a similar nature are available in recent literature, which needs to be cited here

Response by authors: Many thanks. We accept your comments and it is done as per your comments

Comments 8. References 31, 33-34, 38, 42-44 are pretty old references, which need to be replaced by recent, up-to-date citations with better confirmations in these regards.

Response by authors: Many thanks. We accept your comments and it is done as per your comments

Attachment Submitted filename: Point by point response to reviewers.docx

# Editor report: Decision Letter 2

**DOI:** 10.1371/journal.pone.0356876.r005

**Authors:** Ying Ma

10 Aug 2026

Cropland type shapes arbuscular mycorrhizal fungi (AMF) population density and species diversity in Northwest Ethiopia

PONE-D-26-14820R2

Dear Dr. Beyene,

We’re pleased to inform you that your manuscript has been judged scientifically suitable for publication and will be formally accepted for publication once it meets all outstanding technical requirements.

Within one week, you’ll receive an e-mail detailing the required amendments. When these have been addressed, you’ll receive a formal acceptance letter and your manuscript will be scheduled for publication.

An invoice will be generated when your article is formally accepted. Please note, if your institution has a publishing partnership with PLOS and your article meets the relevant criteria, all or part of your publication costs will be covered. Please make sure your user information is up-to-date by logging into Editorial Manager at Editorial Manager® and clicking the ‘Update My Information' link at the top of the page. For questions related to billing, please contact billing support.

If your institution or institutions have a press office, please notify them about your upcoming paper to help maximize its impact. If they’ll be preparing press materials, please inform our press team as soon as possible -- no later than 48 hours after receiving the formal acceptance. Your manuscript will remain under strict press embargo until 2 pm Eastern Time on the date of publication. For more information, please contact onepress@plos.org.

Kind regards,

Ying Ma, Ph.D.

Academic Editor

PLOS One

Additional Editor Comments (optional):

Reviewers' comments:

# Editor report: Acceptance letter

**DOI:** 10.1371/journal.pone.0356876.r006

**Authors:** Ying Ma

PONE-D-26-14820R2

PLOS One

Dear Dr. Beyene,

I'm pleased to inform you that your manuscript has been deemed suitable for publication in PLOS One. Congratulations! Your manuscript is now being handed over to our production team.

At this stage, our production department will prepare your paper for publication. This includes ensuring the following:

* All references, tables, and figures are properly cited

* All relevant supporting information is included in the manuscript submission,

* There are no issues that prevent the paper from being properly typeset

You will receive further instructions from the production team, including instructions on how to review your proof when it is ready. Please keep in mind that we are working through a large volume of accepted articles, so please give us a few days to review your paper and let you know the next and final steps.

Lastly, if your institution or institutions have a press office, please let them know about your upcoming paper now to help maximize its impact. If they'll be preparing press materials, please inform our press team within the next 48 hours. Your manuscript will remain under strict press embargo until 2 pm Eastern Time on the date of publication. For more information, please contact onepress@plos.org.

You will receive an invoice from PLOS for your publication fee after your manuscript has reached the completed accept phase. If you receive an email requesting payment before acceptance or for any other service, this may be a phishing scheme. Learn how to identify phishing emails and protect your accounts at https://explore.plos.org/phishing.

If we can help with anything else, please email us at customercare@plos.org.

Thank you for submitting your work to PLOS One and supporting open access.

Kind regards,

PLOS ONE Editorial Office Staff

on behalf of

Dr. Ying Ma

Academic Editor

PLOS One
